# Study on Functionality and Surface Modification of a Stair-Step Liquid-Triggered Valve for On-Chip Flow Control

**DOI:** 10.3390/mi11070690

**Published:** 2020-07-16

**Authors:** Xi Chen, Sihui Chen, Yi Zhang, Hui Yang

**Affiliations:** 1 Laboratory of Biomedical Microsystems and Nano Devices, Bionic Sensing and Intelligence Center, Institute of Biomedical and Health Engineering, Shenzhen Institutes of Advanced Technology, Chinese Academy of Science, Shenzhen 518055, China; xi.chen@siat.ac.cn (X.C.); sh.chen@siat.ac.cn (S.C.); 2 Center for Medical AI, Institute of Biomedical and Health Engineering, Shenzhen Institutes of Advanced Technology, Chinese Academy of Sciences, Shenzhen 518055, China; 3 CAS Key Laboratory of Health Informatics, Shenzhen Institutes of Advanced Technology, Chinese Academy of Sciences, Shenzhen 518055, China

**Keywords:** capillary microfluidics, liquid-triggered valve, contact angle, surface functionalization

## Abstract

Distinctive from other forms of microfluidic system, capillary microfluidics is of great interest in autonomous micro-systems due to its well-engineered fluidic control based on capillary force. As an essential component of fluidic control in capillaric circuits, micro-valves enable sequential fluidic operations by performing actions such as stopping and triggering. In this paper, we present a stair-step liquid-triggered valve; the functionality of the valve and its dependencies on geometry and surface modification are studied. The surface contact angle of the microfabricated valves that are coated by polyethylene glycol (PEG) or (3-Aminopropyl) triethoxysilane (APTES) is evaluated experimentally, and the corresponding reliability of the valve structure is discussed. Moreover, the variation in the surface contact angle over time is investigated, indicating the shelf time of the device. We further discuss the overall fluidic behavior in such capillary valves, which benefits the capillaric circuit designs at the initial stage.

## 1. Introduction

Microfluidic devices offer the promise of rapid, miniaturized, and automated biochemical assays, as they have potential to realize large-scale applications at a much smaller scale in order to reduce instrument size and sample consumption. Microfluidic devices have been suggested as powerful tools for a variety of applications, including point-of-care diagnosis and bio analysis [1,2,3].

The control of liquid flow at the micro scale is the key to microfluidic applications. Operations of most microfluidic devices require complex peripheral equipment to effect and control the flow of liquid, which in reality limits their scenarios [4]. Governed by capillary effects, capillary microfluidics offer a solution for self-powered fluidic control to get rid of external peripheral instruments. Capillary effects are decided by the interplay between the surface tension of a liquid, the channel geometry, and the surface hydrophilicity—i.e., the surface chemistry of its solid support [5,6,7]. There have been plenty of studies on capillary microfluidic devices with different materials and fabrication methods: paper-based [8,9,10,11,12], polymer-based [13,14], and 3D-printed devices [15], etc. The recent studies of Delarmarche and colleagues have highlighted the potential of silicon-based capillary-driven devices, given their robust fabrication, good chemical resistance, and versatile surface coating methods [16,17]. Hence, it is still favorable, especially in research areas.

A valve is a critical component in the capillary microfluidic system, since it controls the stoppage and resuming of fluidic flow [18]. Essential to most biochemical assays, one could achieve sequential fluidic operations in a capillary system by utilizing different valve structures and arrangements. The features and characteristics of the capillary valves were previously reviewed by Oh et al. [19] and Au et al. [20]. In general, we can classify capillary valves into two groups by their physical natures: local changes of the liquid-vapor surface contact angle and local changes of the surface geometry. In the first scenario, the local change of the contact angle can be regarded as the change of the surface interface that will change the pressure needed to push the liquid along the microchannels [21,22]. In such circumstances, the liquid is usually stopped by hydrophobic patches deposited on a hydrophilic surface, which requires additional patterning and coating procedures. The second type relies on abrupt geometric expansions, which make it energetically unfavorable for the liquid medium to pass through [23,24]. Such geometric valves can then be triggered by an additional flow through a wider channel at the expansion end. The original one-level valve structure via a large expansion angle is often reported to be unstable and can only hold fluid stoppage for short durations (e.g., 5 min [25,26]). Improved valve reliability can be obtained by using a two-level structure with geometric expansions of both the horizontal and vertical interfaces. Zhang et al. proposed a two-level configuration with channels of the same dimension and a stair-step pit at the trigger intersection. The stair-step valve is more versatile and robust and can maintain uniform fluidic characteristics and manifold controls in complex capillary circuits [18].

Capillary pressure is essential in designing the valve geometry, as it has to be sufficient for the liquid to pass the valve for triggering. On the other hand, it cannot break the pressure barrier of the expansions to stop the liquid, otherwise the valve would fail in the first place. The Young–Laplace equation for a rectangular microchannel denotes the close relations among the capillary pressure, channel geometry, and contact angle [27]. In other words, the configuration of surface wettability should be cautiously designed in accordance with the valve geometry to ensure functional stoppage/trigger actions.

Surface modification is considered the most commonly used technique to alter the wettability of capillary microfluidic circuits. Surface tension and wettability vary largely with the surface itself (materials, geometries, etc.), as well as the molecules/chemicals used to modify the surfaces [28]. To develop on-chip immunosensors, the device surface is usually functionalized by coating organic molecules to covalently link biomolecules. The surface properties of traditional inorganic substrates—i.e., silicon and glass—are usually easy to modify; the approach normally includes surface silanization followed by anchoring antibodies to a functional group of the silanizing agent. Furthermore, the efficiency of the surface coverage has an impact on the stability of the surface wettability [29]. As immobilization on planar surfaces yields a limited anchoring point density, the appropriate concentration of functional groups should be employed so as to increase the protein capture capability, resulting in improved immunoassay sensitivity or enzyme conversion rates in immunosensors, for example [30,31,32,33]. Based on the different purposes of microfluidic devices, especially those for diagnostic applications, there are the considerations of whether it needs to lower the non-specific protein absorption or provide anchor points for antibody grafting. Polyethylene glycol (PEG) [34,35,36] and (3-Aminopropyl) triethoxysilane (APTES) [37,38,39] are the most representative silanizing agents that have been extensively employed for the purposes above.

When the liquid trigger valve is fabricated on silicon substrate, the superhydrophilic nature of the bare Si surface provides an overwhelming capillary pressure against the energy barrier of the expansion geometry in the stop flow channel [40]. This could lead to a complete flow-through action without the need of a secondary trigger flow, meaning the failure of the valve structure. The PEG and APTES treatments both render a moderate surface hydrophilicity to ensure the high yield of the functional capillary valve. However, the modified surfaces are commonly subject to changes during storage and experience a decrease in surface hydrophilicity. This phenomenon is called hydrophobic recovery [41,42]. The resulting contact angle will increase over time and inevitably alter the fluidic dynamics against the design’s original intention, hence it could lead to the failure of the capillary fluidic or the misconduct of downstream bioassays. While rarely discussed in previous works, researchers cannot afford to ignore the aging effect of the surface functional groups, given that the stability of products is essential in practical use, for example in point-of-care applications.

In this paper, we present a stair-step liquid-triggered capillary valve on a silicon substrate that is designed to work with a closed-top surface. The nature of enclosed microfluidics can prevent liquid evaporation and lower the risk of contamination across the system [7,43]. Following with photolithography and dry etching processes, we evaluate the functionality of such a valve based on its ability to stop/trigger fluidic flow under different surface conditions. In this study, a one-step surface modification method is presented to prepare the silicon microfluidic devices with either PEG or APTES in an aqueous solution. We discuss their influences on valve functionality, and the stability of different surface functional groups is evaluated as well.

## 2. Materials and Methods

### 2.1. Chip Design and Fabrication Process

**Figure 1:**
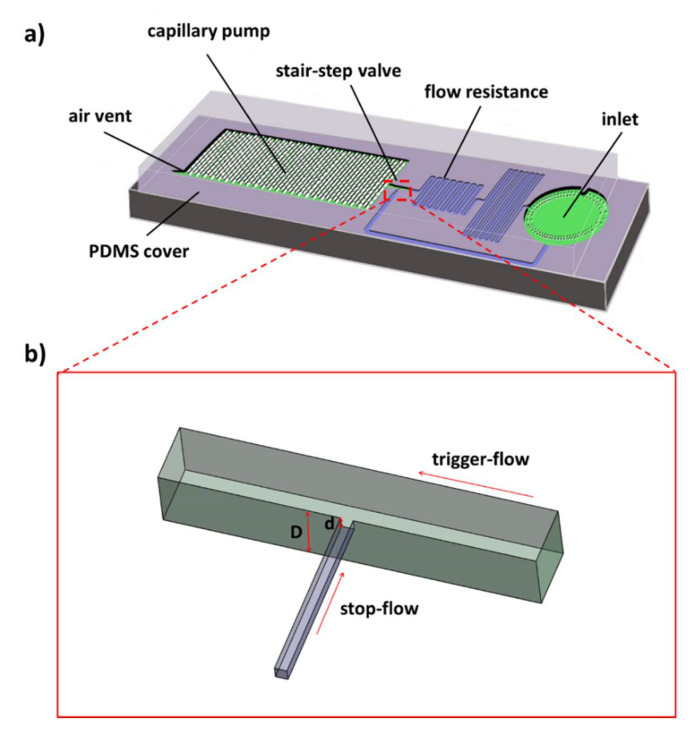
Schematic illustration. (**a**) The microfluidic device; (**b**) the stair-step liquid-triggered valve.

The microfluidic chip with length of 10 mm and width of 4 mm consists of a silicon substrate with microfluidic structures and a thin film of Polydimethysiloxane (PDMS, Sylgard 184, Dow Corning GmbH, Wiesbaden, Germany) as the top layer to enclose the microfluidic channels (Figure 1a). The silicon substrate includes one inlet, a flow resistance to tune the flow speed and also control the sequence of fluidic operations, a stair-step liquid-trigger valve, as well as a capillary pump and an air vent. The valve structure is shown in Figure 1b in detail. It consists of a stop-flow microchannel that is generated by a shallow etch process, and a trigger-flow microchannel made by a deep etch process is put perpendicularly to the stop-flow channel to generate the stair. The sharp edges at the stair create an energy barrier to pin the liquid meniscus. It should also be noted that the upstream channel dimensions determine the mixing ratio of the liquid media from the stop-flow and the trigger-flow channels. Therefore, the flow resistance of the trigger-flow channel is much larger than that of the stop-flow channel, as it is required in most applications that the triggering action needs to be realized with the minimum dilution of the stopped fluid.

**Figure 2:**
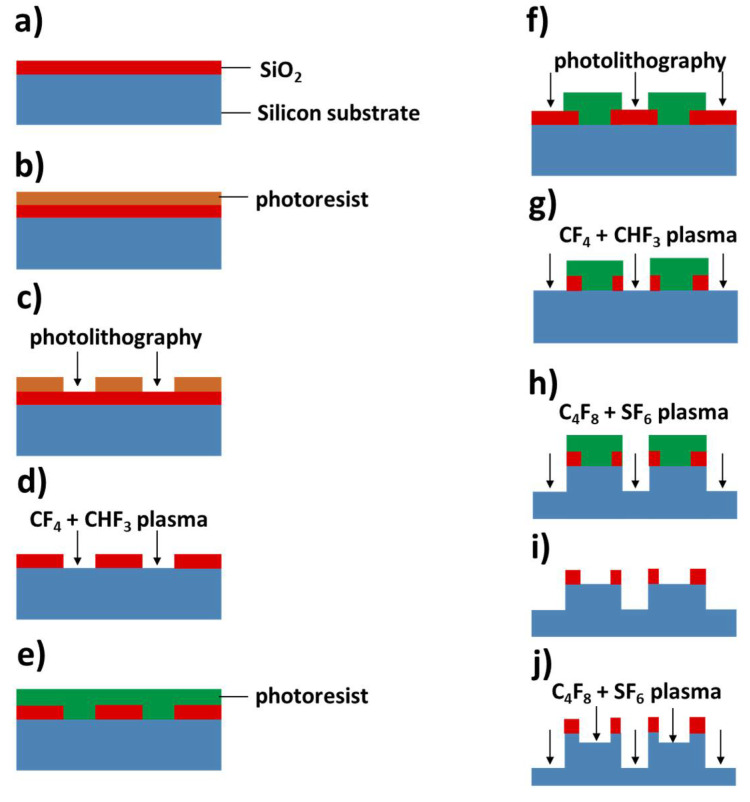
Schematic illustration of the microfabrication process. (**a**) SiO_2_ is deposited on the silicon wafer; (**b**,**c**) photoresist is spin-coated on the wafer and patterned by a standard photolithography process; (**d**) SiO_2_ is etched by a CF_4_ + CHF_3_ plasma using RIE; (**e**,**f**) a thick photoresist layer is spin-coated on the wafer and patterned by a standard photolithography process; (**g**) SiO_2_ is etched by a CF_4_ + CHF_3_ plasma using RIE; (**h**) the silicon is etched by C_4_F_8_ + SF_6_ plasma using DRIE; (**i**,**j**) the photoresist is stripped and the silicon is etched by DRIE.

The silicon chip is fabricated by a two-step silicon etching process. The microfabrication process flow is shown in Figure 2. Silicon wafer (Ø4 in., 450 μm in thickness) is firstly coated by a thin layer (200 nm in thickness) of SiO_2_ that is made by thermal oxidation and used as the hard mask for the upcoming silicon etching processes. This hard mask is then patterned by standard photolithography using the AZ1500 positive photoresist (0.6 μm thickness, MicroChemicals GmbH, Ulm, Germany) and a mask aligner 610TB (EV Group GmbH, St. Florian am Inn, Australia), following by reactive ion etching (RIE) with CF_4_ + CHF_3_ plasma (RIE-10NR, Samco Inc., Kyoto, Japan) to open the window for shallow silicon etching. After removing the residual AZ 1512 layer, the substrate is then patterned by a second photolithography process using the AZ4620 positive photoresist (10 μm thickness), followed by RIE CF_4_ + CHF_3_ plasma etching to open the window for silicon deep etching on the SiO_2_ layer. Deep RIE (DRIE) with C_4_F_8_ + SF_6_ plasma (DSE200S, NAURA Technology Group Co., Ltd., Beijing, China) is used to etch the silicon substrate for a depth of 105 µm first. Then the remaining photoresist layer is stripped, leaving the window for silicon etching fully open. As the last step, the silicon substrate is etched for an additional 25 µm of thickness. In such way, the designated depths of the shallow and the deep layer of the microfluidic system are 25 and 130 µm, respectively, which will be further discussed in detail in Section 3.1.

### 2.2. Surface Modification of the Silicon Chip

**Figure 3:**
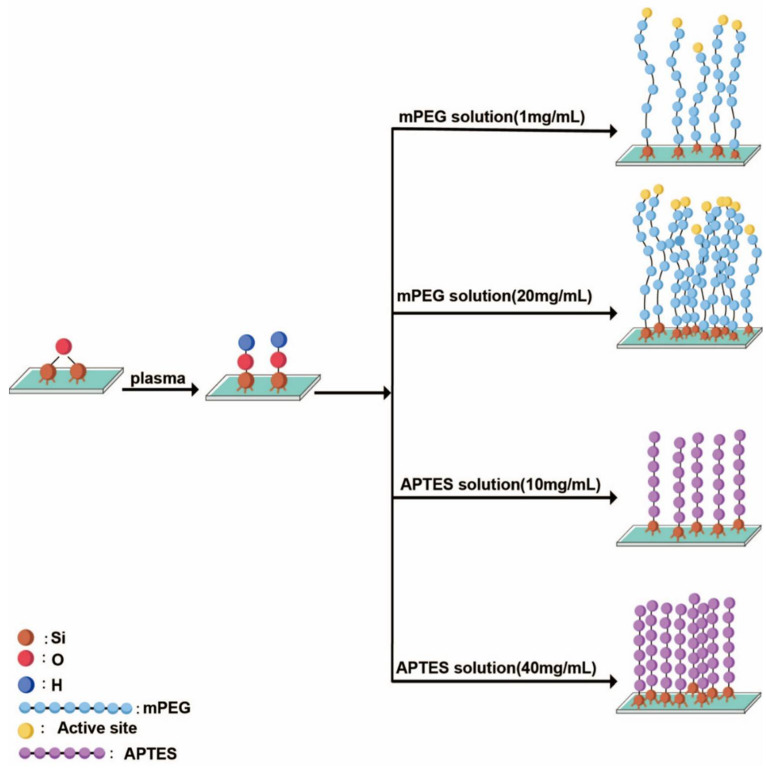
Schematic illustration of surface modification methods, showing the differences in the mPEG and APTES grafting density on the chip surface.

After the fabrication of the microfluidic systems, the silicon chip is functionalized by either PEG or APTES. PEG is often used to prevent bio-fouling by minimizing the unspecific absorption of small molecules on the surface of the device, and APTES is usually utilized to generate silane patterns in the microfluidic channels for protein immobilization [44]. The silicon chips are firstly exposed to oxygen plasma for 3 min at room temperature and atmospheric pressure. This step cleans the surface of the device and generates silanol groups (Si-OH) for a subsequent surface treatment procedure (Figure 3). The surface functionalization is applied by immersing the chips overnight in either mPEG (molecular weight: 3400, Renbang Pharmaceutical Technology Co. Ltd., Shanghai, China) solutions of 1 and 20 mg/mL, or APTES (molecular weight: 221, Sigma-Aldrich, St. Louis, MO, USA) solutions of 10 and 40 mg/mL, respectively. The treated chips are then cleaned by ultrasonication in toluene and isopropyl alcohol (IPA), subsequently, and then baked in a vacuum for 1 h. Regarding the surface density of the functional groups, previous works have investigated the relationship between the different reaction factors and the quality of the surface coating. When the molecule weight of mPEG ranges from 2100 to 5400, the grafting density can vary from 2.4 to 3.9 molecules per square nanometer, generating a layer of few tens of nanometer [45]. The APTES density on glass surfaces is on average between 2.1 and 4.2 amine groups per square nanometer [46,47]; the thickness of the functional layer can vary under certain conditions, from a few to tens of nanometers [48].

### 2.3. Experimental Method and Setup 

**Figure 4:**
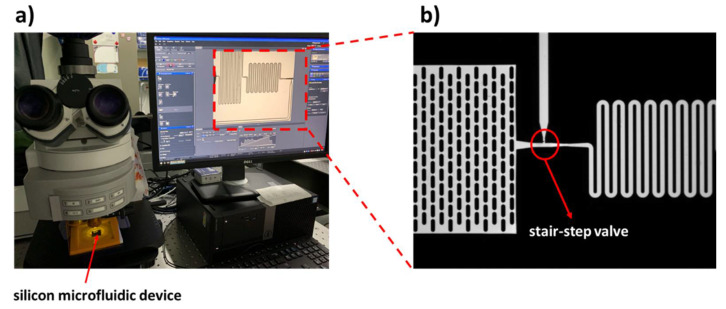
The experimental setup for optical inspections of the fluid flow in the microfluidic device. (**a**) An upright optical microscope equipped with a fluorescent light source; appropriate filter sets and a CCD camera is used to inspect the fluid behavior. (**b**) The test result is clearly observed inside the microfluidic structure with the Rhodamine B solution.

An optical contact angle measurement tool (DSA25, Krüss GmbH, Hamburg, Germany) is used to determine the equilibrium contact angles of water on the chip surface, which is obtained by averaging five measurement results at different locations on each chip. The experimental setup is shown in Figure 4. Optical inspections of the fluid flow in the microvalve structures are performed using an Axio Imager M2p (Carl Zeiss Microscopy GmbH, Jena, Germany) upright fluorescent microscope (see Figure 4a) equipped with a 20× objective with numerical aperture of 0.4 (Zeiss Objective LD Plan-Neofluar 20×/0.4 Corr M27, 421350-9971-000). The microscope is configured with a mercury vapor short arc lamp (X-Cite 120Q, Carl Zeiss Microscopy GmbH, Jena, Germany) and appropriate fluorescent filter sets for illumination and signal detection. Image acquisition is performed using a CCD camera (Axiocam506 mono, Carl Zeiss Microscopy GmbH, Jena, Germany). The chips are tested by using deionized water mixed with a fluorescence dye, Rhodamine B (Sigma Aldrich, St. Louis, MO, USA), at a concentration of 100 µg/mL. The mixture has a contact angle similar to pure water. The silicon chip is covered by a thin PDMS film to enclose the fluidic channels. The fluid situation in the microfluidic chip can be clearly observed using the Rhodamine B solution (as shown in Figure 4b).

## 3. Results and Discussion

### 3.1. Fabrication and Topography of the Valve

**Figure 5:**
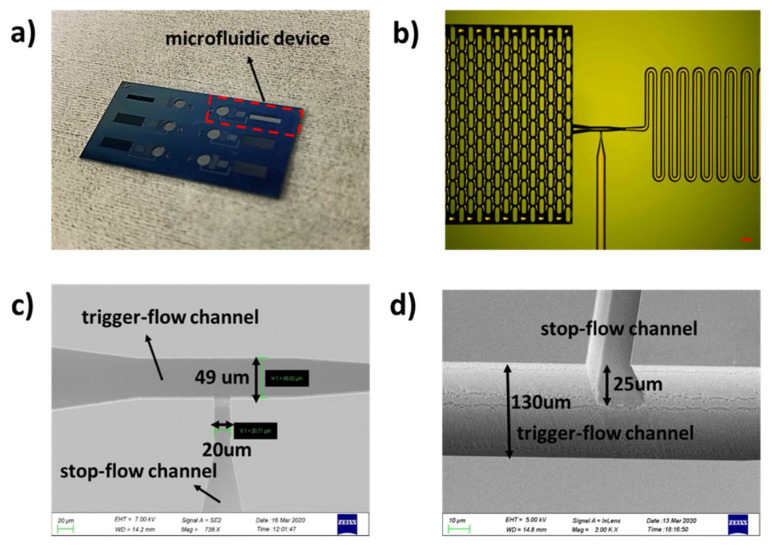
The microfluidic device fabricated by the two-step silicon etching process. (**a**) Image of the chip with six valve structures. The red dotted area is one microfluidic system for testing; (**b**) microscopic image of the silicon microfluidic device, scale bar is 100 µm. (**c**,**d**) SEM images of the fabricated valve. The valve structure consists of a stop-flow channel and a trigger-flow channel; (**c**,**d**) the channel widths and depths, respectively.

The microstructure is characterized after the microfabrication process. As shown in Figure 5a, there are six microfluidic systems with the identical valve structures on a single chip, and Figure 5b is the optical microscopic image showing the microfluidic structures. The topography of the microvalve is characterized by a scanning electron microscope (SEM, Gemini, Carl Zeiss Microscopy GmbH, Jena, Germany). Figure 5c,d show the measurements of the microvalve; the stop-flow channel is 20 μm in width and 25 μm in depth, while the trigger-flow channel is 49 μm in width and 130 μm in depth after microfabrication. It should be noted that the choices of the valve dimension are determined by the combination of the technical limitations of microfabrication and the design requirements of a complete fluidic circuit. The widths of the stop-flow channel and the trigger-flow channel are chosen mainly due to the fluidic impedance of the upstream fluidic circuit. In microfabrication processes on a silicon substrate, one can usually obtain two-step structures by lithographic techniques and DRIE, and it is possible to obtain an aspect ratio of 30 [49]. During such procedures, precise and deep structures with vertical sidewalls, sharp edge features, and high aspect ratios are necessary for robust stair-step capillary valves. Therefore, the depth of 130 μm for the deep etch is determined after considering these requirements; moreover, so are the fabrication aspect ratio and the smallest structure on the microfluidic circuit—i.e., the micro-pillar structures in the capillary pump. Besides this, according to our practice, the aspect ratio of the deep and the shallow etch should be larger than 5:1 to obtain a robust valve so that the liquid meniscus can be pinned at the sharp edges created at the interfaces of the two depths.

In our design, a geometric expansion of the channel cross-section along both the channel width and depth is used to generate a robust valve structure. It should be noted that a sharp transition between the two steps is essential, because a smooth transition could cause the valve to leak. Therefore, the two-step DRIE process presented in Section 2.1 is used in the fabrication. Usually, in order to etch two different depths on one wafer, one could try to spin-coat the photoresist needed for the second etch on top of the structures generated by the first etching step. However, when the depth of the first etch step is high enough, it is very difficult to deposit a resist layer with a uniform thickness for standard photolithography. Therefore, we present such a process flow that the thermal oxide layer is firstly patterned for the shallow features, but instead of the DRIE process to etch silicon, a second photolithography with a thick resist layer is performed subsequently. Considering that the thickness of the thermal oxide is only 200 nm, the uniformity on the resist thickness is secured. Then, the deep features are etched, using the thick resist layer as the hard mask for the first step of 105 µm. After this step, the residual resist is thoroughly removed from the surface, leaving all the windows open for DRIE, and the second DRIE step is performed to etch another 25 µm, generating a two-step structure with depths of 25 and 130 µm, respectively. Such process flow promises sharp transitions between the two steps, and the results obtained from SEM characterization are shown in Figure 5. 

### 3.2. Surface Functionalization Results

**Figure 6:**
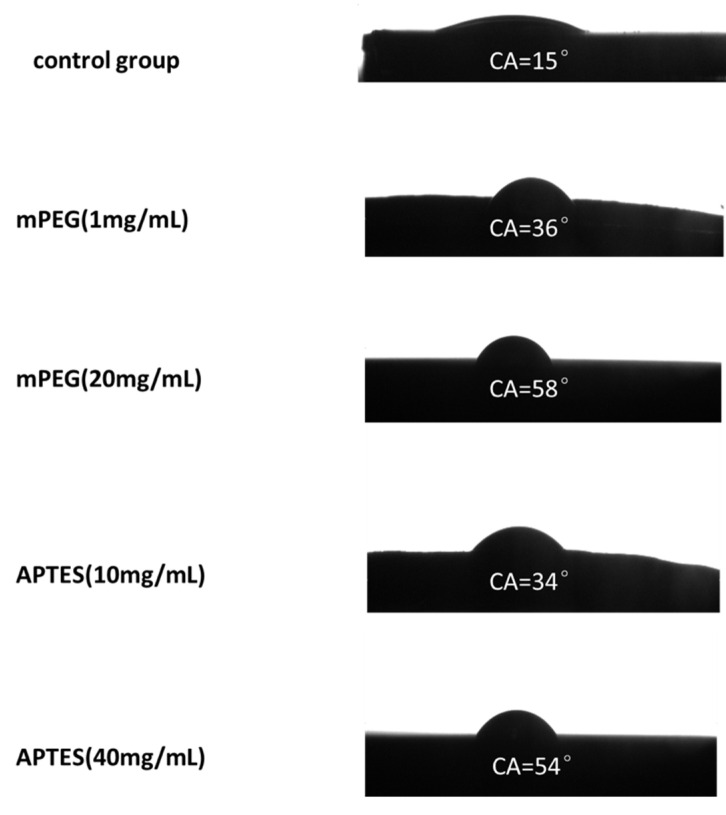
Contact angle measurement results on chip surfaces that are modified with different conditions. The left column shows the modification conditions, and the right column the corresponding contact angles.

Following the device fabrication, the chip surface is functionalized either by mPEG or by APTES, and the equilibrium static contact angle is measured at different locations on the chip surface to characterize the wetting property of the device. The results are shown in Figure 6. The chips functionalized by mPEG with concentration of 1 mg/mL show a contact angle of 36° ± 0.8° (mean ± standard deviation). When the mPEG concentration increases to 20 mg/mL, the contact angle also increases to a valve of 58° ± 0.8°. Furthermore, when the chips are treated by APTES, the contact angles are measured as 34° ± 0.8° and 54° ± 0.8° using the APTES solutions of 10 and 40 mg/mL, respectively. As a comparison, the static contact angle is measured as 14° ± 0.8° when the device surface is not functionalized.

**Figure 7:**
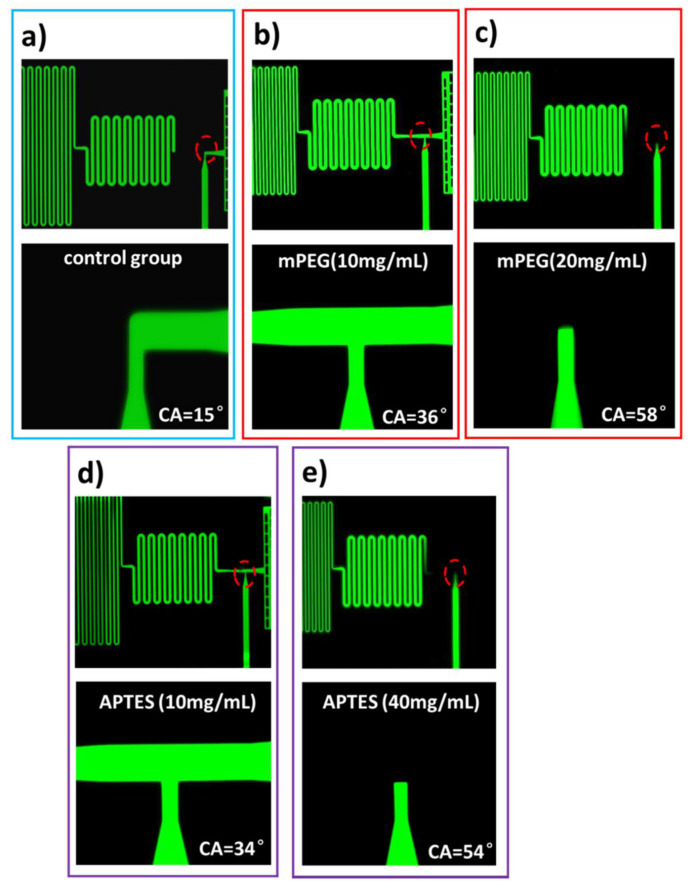
Test results of valve functionality at five different contact angles. (**a**) For the valve with contact angle of 15°, the fluid from the stop-flow channel leaks into the trigger-flow channel; (**b**) chips treated by 1 mg/mL mPEG show a contact angle of 36° and the valve is fully functional; (**c**) chips treated by 20 mg/mL mPEG show a contact angle of 58°, the fluid stops in the stop-flow channel but is not triggered afterwards; (**d**) chips treated by 10 mg/mL APTES show a contact angle of 34° and the valve is fully functional; (**e**) chips treated by 40 mg/mL APTES show a contact angle of 54°, the fluid stops in the stop-flow channel but is not triggered afterwards. (**a**–**e**) The structures in the red dash-dot lines are enlarged in the second row of the figure.

The functionality of the valves with different surface modifications are then evaluated by using a solution of Rhodamine B in deionized water with a concentration of 100 µg/mL. Six devices are tested at each surface condition. The experimental results are consistent, and the results obtained from different conditions are shown in Figure 7. For the chips free of any surface coatings, the valve with a surface contact angle of ~15° results in leakage immediately when the fluid from the stop-flow channel reaches the junction and escapes into the trigger-flow channel (Figure 7a). When the silicon chip is coated with an mPEG of 1 mg/mL, the surface contact angle of 36° ensures that the fluid stops in the stop-flow channel, as the abrupt geometric expansion makes passing through such a structure energetically unfavorable (Figure 7b). For the chips coated with an mPEG of 20 mg/mL, the higher contact angle (58°) makes the valve structure more robust. However, it takes much longer for the fluid to pass the flow resistance, and the elevation of flow resistance stops the fluid before it reaches the valve (Figure 7c). The test results indicate that there is a working range of the contact angle, which should be high enough, but its influences on the flow speed over the full system need to be evaluated carefully. A similar phenomenon is observed when the chip surface is modified by APTES solutions. The 10 mg/mL APTES generates a contact angle of 34° on the chip surface; the fluid stops in the stop-flow channel and is then triggered by the fluid coming from the trigger-flow channel (Figure 7d). While the concentration of APTES increases to 40 mg/mL, the contact angle of 54° is too high to pass the flow resistance, and hence the valve cannot be triggered properly (Figure 7e).

### 3.3. Characterization on the Shelf Time of Different Surface Functionalization Groups

**Figure 8:**
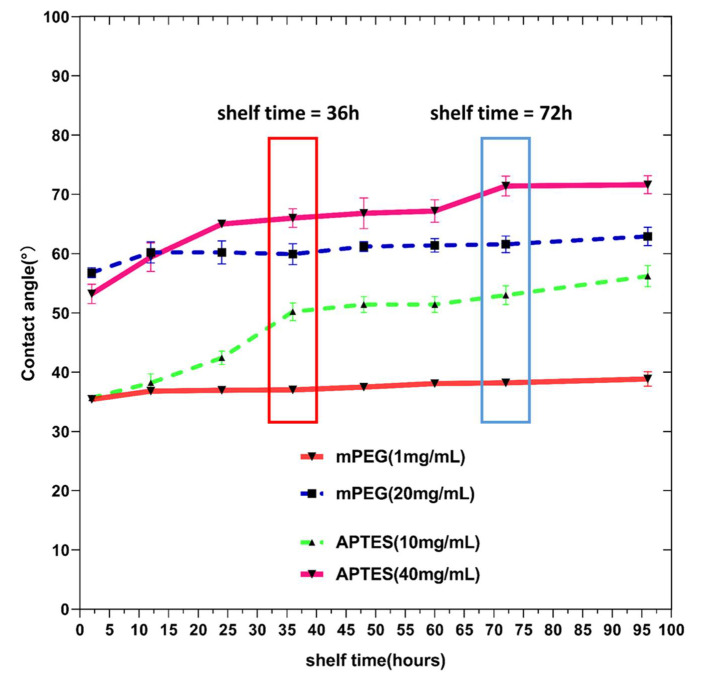
Contact angle measurements as a function of shelf time. Dots in the figure indicate the average of 10 tests on two chips, and the error bars indicate the standard deviation.

In close relation to the functionality of silicon capillary microfluidic chips, the shelf time of the different mPEG/APTES treatment groups on the contact angle point-of-view is further assessed experimentally. The contact angle on the device surface at different times and the corresponding valve functionality are tested. As shown in Figure 8, the contact angles of the surfaces modified by both mPEG and APTES increases with time, and mPEG is more stable than APTES, as the contact angles obtained from mPEG treatment groups show minor increases over time comparing to that from the APTES treatment groups. It is found that the contact angle increases less than 3° and 5° after 4 days for the devices treated by mPEG with concentrations of 1 mg/mL and 20 mg/mL, respectively. However, when the devices are treated by APTES, their surface contact angles tent to increase rapidly in the first 24 h. From the polymer science aspect, the difference on the time course of the contact angle between polymers can be mainly due to surface configuration change [42]. When exposing to atmosphere, the reactive aminoalkoxyslanes groups show less resistance against low molecular weight oxidized materials compared to the methoxy groups of the mPEG end. The oxidation and hydrolysis of amine trigger attacks on the reactive sites of other APTES molecules or the Si substrate. Such horizontal reaction reorients hydrophilic amine groups much closer to the substrate, making the contact angle increase [42,50,51]. Besides, comparing to the small molecular chain of APTES, the flagellum-like mPEG long chain makes the reorientation of hydrophilic groups energetically unfavorable. Therefore, the mPEG-modified surface is capable of prohibiting hydrophobic recovery, showing a better stability in the contact angle.

**Figure 9:**
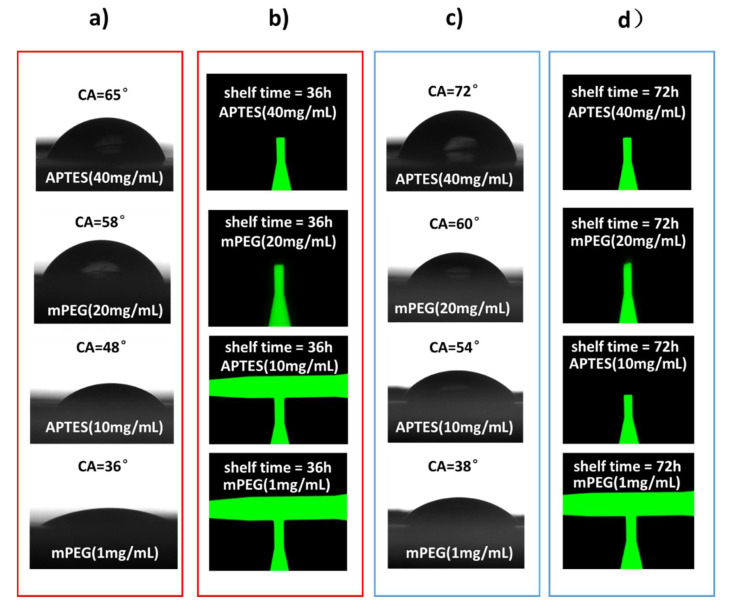
Valve functionality in relation to the shelf time with different surface modification methods. (**a**) Measurement results on the surface contact angle of chips 36 h after they are treated by APTES (40 mg/mL), mPEG (20 mg/mL), APTES (10 mg/mL), and mPEG (1 mg/mL), respectively; (**b**) evaluation of the feasibility of valves with surface functional groups obtained from (**a**); (**c**) measurement results of the surface contact angle of chips 72 h after they are treated by APTES (40 mg/mL), mPEG (20 mg/mL), APTES (10 mg/mL), and mPEG (1 mg/mL), respectively; (**d**) evaluation of the feasibility of valves with the surface functional groups obtained from (**c**).

As shown in Figure 9, the functionality of the valves is tested at each time point and the surface treatment group with two chips; the experimental procedure is consistent. As the inserts showing the results at 36 h and 72 h in Figure 9, the valves can be triggered under the mPEG (1 mg/mL) treatment with different shelf time conditions. If the valve is treated by APTES (10 mg/mL), it can only be triggered when the shelf time is less than 36 h. All the other devices cannot work properly. Therefore, mPEG is a better solution, considering the stability of the surface functional groups.

## 4. Conclusions

Liquid-triggered micro-valves are of great interest in capillary-driven planar microfluidic systems. In this paper, we present a stair-step liquid-triggered valve and test its functionalities in capillary circuits. In this study, the valves with geometric expansions in both horizontal and vertical interfaces are fabricated by a two-step DRIE process on silicon substrate. In particular, a process flow that can ensure sharp transitions on the interfaces of the stop-flow channel and the trigger-flow channel is proposed. The surface of the microfluidic chips is functionalized by mPEG and APTES of different concentrations; the equilibrium contact angle and its variation over time are evaluated. In order to have a functional valve, not only the liquid from the stop-flow channel should stop at the interface of shallow and deep channels, but also the triggering liquid should flow through the trigger-flow channel. Therefore, mPEG and APTES should maintain proper concentrations for device surface modification to keep the contact angles in a working range from 30° to 50°. The experimental results show that mPEG-modified surfaces present a better stability over time, with the concentration of 1 mg/mL in the surface treatment solution excelling in valve performance. Yet the reactive amine groups and their short molecular chain nature make APTES a choice for rather short-term use. When designing a complex capillaric circuit for long-term applications, it is essential to consider all the combining factors, including channel geometry, surface contact angle, as well as the shelf time of surface functioning groups. We believe this article would provide a guideline for integrating a robust trigger valve structure into capillaric systems designed for various purposes.
